# Impact of sex and sex hormones on pathophysiology and progression of aortic stenosis in a murine model

**DOI:** 10.14814/phy2.15433

**Published:** 2022-08-27

**Authors:** Marie‐Ange Fleury, Mohamed‐Salah Annabi, Martine Voisine, Maxime Hervault, Anne‐Julie Boilard, Mylène Shen, André Marette, Nancy Côté, Marie‐Annick Clavel

**Affiliations:** ^1^ Institut universitaire de cardiologie et de pneumologie de Québec‐Université Laval / Québec Heart and Lung Institute, Université Laval Québec city Canada

**Keywords:** aortic stenosis, pathophysiology, sex differences

## Abstract

The lesions observed in AS have been shown to be sex specific, with women presenting extensive fibrotic remodeling while men developing more calcification deposit. We thus aimed to evaluate the influence of sex and sex hormones on the pathophysiology of aortic valve stenosis (AS) in our mouse model of AS. LDLr^−/−^ApoB^100/100^IGF‐II^+/−^ mice (*n* = 210) were separated in six different groups: (1) intact male (IM), (2) intact female (IF), (3) castrated male (CM), (4) ovariectomized females (OF), (5) CM with testosterone supplementation (CMT), and (6) OF with 17β‐estradiol supplementation (OFE). Mice were fed a high‐fat/high‐sucrose/high‐cholesterol diet for 6 months. Hemodynamic progression of AS was followed by transthoracic echocardiography (at 12 and 36 weeks) and analyzed in all mice alive at 36 weeks. Aortic valves were collected for histological and digital droplet PCR* analysis. Increases in peak velocity were comparable in IF and IM (24.2 ± 5.7 vs. 25.8 ± 5.3 cm/s; *p* = 0.68), but IF presented with less severe AS. Between the three groups of male mice, AS progression was more important in IM (increase in peak velocity: 24.2 ± 5.7 cm/s; *p* < 0.001) compared to CM (6.2 ± 1.4; *p* = 0.42), and CMT (15.1 ± 3.5; *p* = 0.002). In the three groups of female mice, there were no statistical differences in AS progression. Digital PCR analysis revealed an important upregulation of the osteogenic gene RunX2 in IM (*p* < 0.0001) and downregulation of the pro‐calcifying gene ALPL in IF (*p* < 0.05). Male sex and testosterone play an important role in upregulation of pro‐calcifying genes and hemodynamic progression of AS. However, female mice appeared to be protected against calcification, characterized by downregulation of pro‐osteogenic genes, but presented a similar AS hemodynamic progression.

## Graphical abstract

The progression rate of aortic stenosis is comparable between male and female, however the composition of the valve associated with this progression is sex specific. Stenosed aortic valve from female mice present a lower degree of calcification with a higher degree of fibrosis compared to the ones from male mice. Testosterone, in male, appear to play a major role in aortic valve calcification and aortic stenosis progression with the fastest progression rate in intact male mice (i.e., with norm testosterone level), an intermediate progression in castrated male mice with partial supplementation in testosterone, and finally no significant progression rate of aortic stenosis in male without testosterone. Pro‐calcific pathways, especially RUNX2, are overexpressed in male mice and by testosterone. Female mice appear to be protected against calcification independently of estrogen level.

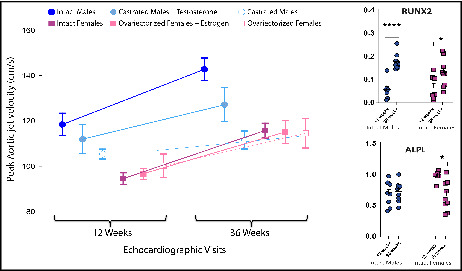

## 1 INTRODUCTION

Fibro‐calcific aortic valve stenosis (AS) is the most common valvular disease and the third most common cardiovascular disease in high‐income countries after coronary artery disease and systemic arterial hypertension. (Nkomo et al., 2006) The incidence of severe AS increases exponentially with age to reach 3 to 4% of the population over 65‐year‐old. (Eveborn et al., 2013; Nkomo et al., 2006) No medical therapies have been proven to slow down the progression of valve stenosis and AS progression rate have been linked to mortality even in asymptomatic patients (Benfari et al., 2020). The only efficient treatment is the replacement of the affected aortic valve by surgical or transcatheter approach. In addition, our poor understanding of how sex affects the pathophysiology of AS considerably limits our ability to personalize management of this disease for both sexes. This is highlighted by the fact that AS pathophysiology has been traditionally studied in cohorts with a large majority of men (Bossé et al., 2008; Côté et al., 2010; El Husseini et al., 2014), given that male sex has been identified as a risk factor for AS. (Freeman & Otto, 2005) Accordingly, animal studies have typically been performed in male animals only in order to reduce heterogeneity. (Bouchareb et al., 2015; Rajamannan et al., 2001) However, we previously showed that women reach similar hemodynamic severity of AS with a lower aortic valve calcification load compared to men. (Aggarwal et al., 2013; Clavel et al., 2013) In addition to aortic valve calcification, lipid infiltration, aberrant extracellular matrix remodeling, and extensive valvular fibrosis may contribute to the thickening and stiffening of the aortic valve leaflets. Women's stenotic valves also present denser connective tissue with a significantly greater relative amount of collagen fibers compared to men's stenotic valves. (Simard et al., 2017; Voisine et al., 2020) Thus, in the present study, we aimed to assess the impact of sex and sex hormones on AS pathophysiology and progression in a murine model of AS.

## 2 METHODS

This study was conducted within the Mouse Animal model of Sex Differences in Aortic Stenosis (MASDAS) study using C57BL6 mice LDLr^−/−^/ApoB^100/100^/IGF‐II^+/−^. These mice develop AS within 36 weeks under a high‐fat/high‐sucrose/high‐cholesterol diet. (Le Quang et al., 2014) This protocol was approved by the Laval University Animal Care and handling Committee.

### 2.1 Mouse model

**FIGURE 1:**
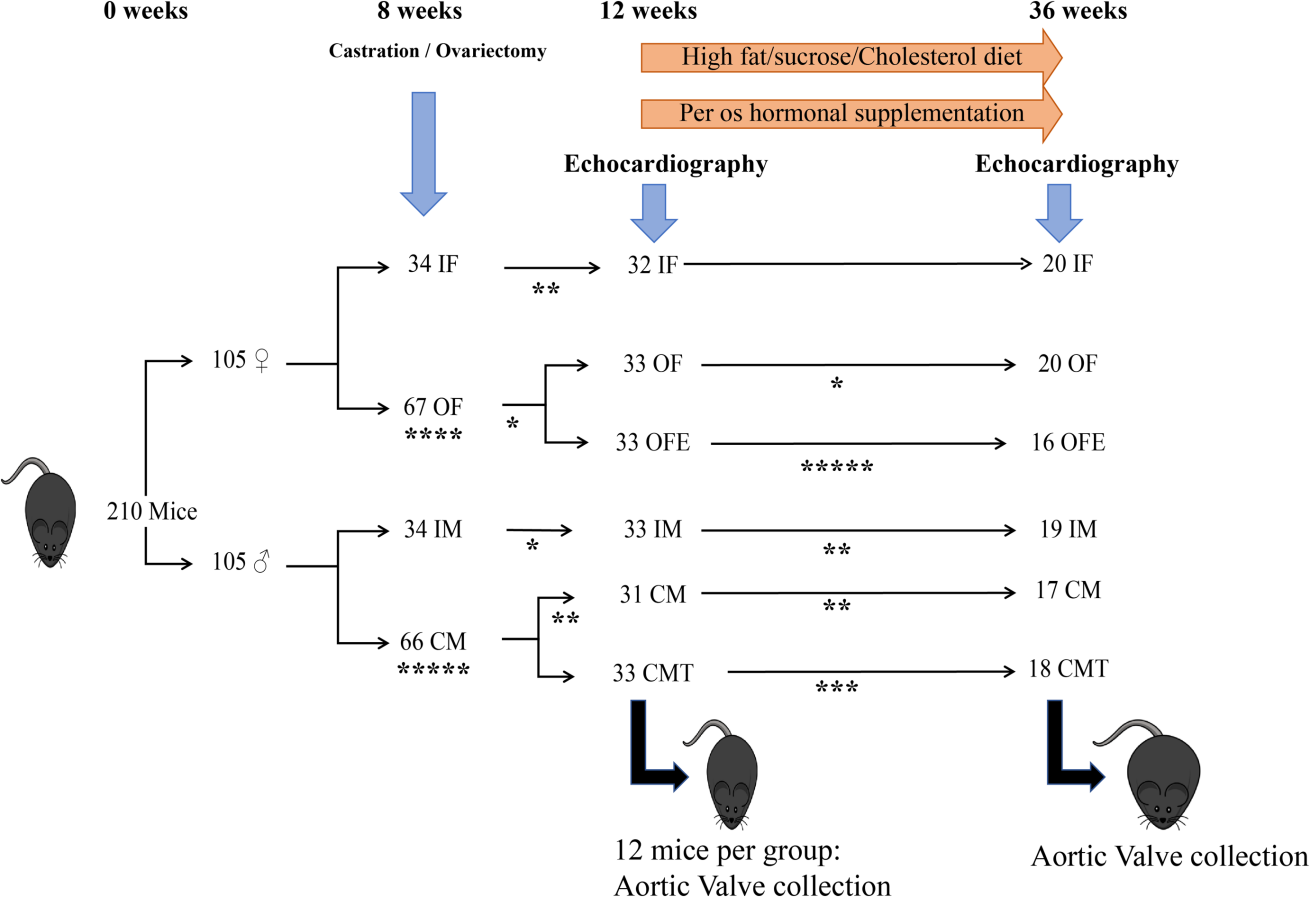
Schema of the protocol. CM, castrated male; CMT, Castrated male with testosterone supplementation; IF, intact female; IM, Intact male; OF, Ovariectomized female; OFE, Ovariectomized female with 17 β estradiol supplementation. *unexpected mortality.

Our LDLr^−/−^ ApoB^100/100^ IGF‐II^+/−^ mice were individually housed in ventilated cages, in a pathogen‐free, temperature‐controlled environment under a 12:12 h light–dark cycle. Up to 12 weeks of age, mice were fed ad libitum with a standard rodent diet. Beyond12 weeks of age a high‐fat/high‐sucrose/high‐cholesterol diet (65% kcal from fat; provided by Envigo*, Indianapolis, USA) was introduced for the remainder of the protocol. At 8 weeks, two thirds of the mice underwent gonadectomy under general anesthesia and at 12 weeks, half of gonadectomized and castrated mice were supplemented with sex hormones according to their sex (i.e., testosterone in males and 17β estradiol in females). Within each sex, mice were randomly assigned to a given group (intact, gonadectomized or supplemented) and all analysis (i.e., echocardiography, histology, and PCR) were performed blinded to the allocation group. With these interventions, six groups of mice were studied: intact males (IM), intact females (IF), castrated males (CM), ovariectomized females (OF), CM with testosterone supplementation (CMT), and OF with estrogen supplementation (OFE) (Figure 1). As daily injection of hormone was not feasible for a 24 weeks study, and pellets of testosterone were not available, we elected to supplement the animals per os. Testosterone (Andriol® 40 mg; Merck Canada: 0.1 mg/day/30 g mouse PO) and 17β estradiol (Sigma‐Aldrich Canada: 1.12 μg/day/30 g mouse PO) were diluted in 0.312 μl of sesame oil (Sigma‐Aldrich Canada) and served every morning to each supplemented mouse in 1 ml Nutella®. Non‐supplemented mice received every morning 0.312 μl of sesame oil in 1 ml Nutella®. The level of hormones was comparable between intact animals and supplemented animals (Appendix S1).

### 2.2 Gonadectomy

Anesthesia was induced in a dedicated chamber with 4–5% isoflurane. A nose cone was used for continuous isoflurane (3%) delivery titrated to maintain stage 4 general anesthesia. Analgesia was performed by long acting buprenorphine 0.05 mg/kg SC and lidocaine (7 mg/kg)/bupivacaine (3.5 mg/kg) SC at the level of the skin incision.

Castration was realized by a scrotal approach. The skin at the center of the scrotum was dissected and the vaginal tunic was exposed. It was then incised, and the testis was exposed. It was then ligated, and the blood vessels were cauterized. The same was done for the second testis. The vaginal tunic was placed back in the scrotum and the skin was then stapled.

Ovariectomy: A posterior incision was made at the last rib over the spinal column. The muscle on one side was dissected and the ovary was exposed. The ovary was ligat removed. The fat and the ovary duct were placed back into the abdominal cavity. The muscle was stitched using absorbent sutures. The same steps were repeated on the other side. The skin was closed using a staple.

### 2.3 Echocardiography

Mice underwent an echocardiographic examination at 12 and 36 weeks of age.

Anesthesia was induced in a dedicated chamber with 4–5% isoflurane. Then the mice were maintained in left lateral decubitus on a heated platform. A nose cone was used for continuous isoflurane (1.5–3%) delivery titrated to maintain stage 2 general anesthesia (pre‐surgical level according to experimental animal anesthesia guidelines (Fish et al., 2008) during image acquisition). Chest and abdominal fur were removed using a hair‐removal cream.

Transthoracic echocardiography was performed with L15‐7io (linear 7–15 Megahertz) and S12‐4 (4–12 Megahertz) probes connected to a Philips HD11XE ultrasound system (Philips Healthcare Ultrasound, The Netherlands) (Annabi et al., 2020). The linear probe was used for left ventricular (LV) M‐mode imaging in parasternal long‐axis view at the basal third of the left ventricle. Left ventricular internal dimensions were measured at end‐diastole and end‐systole and fractional shortening was calculated as $\left(\frac{\text{LVEDD}-\text{LVESD}}{\text{LVEDD}}\right)\times 100$, with LVEDD and LVESD the left ventricular internal dimensions at end‐diastole and end‐systole, respectively.

Continuous‐wave Doppler was used to record peak transvalvular aortic jet velocity (V_peak_) and velocity‐time integral. Left ventricular outflow tract velocities were sampled using pulsed‐wave Doppler. Left ventricular outflow tract velocities were considered optimal when the aortic valve closing click but not the opening click was present (Annabi et al., 2020). The cross‐sectional area of the left ventricular outflow tract was calculated as $\;{\text{LVOT}}_{\mathrm{CSA}}=\pi \times {\left(\frac{{\text{LVOT}}_{D}}{2}\right)}^{2}$, with LVOTD the left ventricular outflow tract diameter.

Then, the left ventricular stroke volume (SV) was calculated in left ventricular outflow tract: ${\text{LVOT}}_{\mathrm{CSA}}\times {\text{LVOT}}_{\mathrm{VTI}}$, with LVOT_VTI_ the left ventricular outflow tract velocity‐time integral.

Aortic valve area was calculated with the continuity equation: $\mathrm{AVA}=\frac{{\text{LVOT}}_{\mathrm{CSA}}\times {\text{LVOT}}_{\text{Vmax}}}{V_{\text{peak}}}$, with LVOT_Vmax_ the maximal velocity in the left ventricular outflow tract and Vpeak the peak transvalvular aortic jet velocity. Then AVA was indexed (AVAi) to the body surface area (calculated as the weight (kg)/3, following a method validated in pharmacologic studies (Dawson, 1967)) and cardiac output was calculated as heart rate × SV and indexed to body surface area (CI).

### 2.4 Aortic valve histology

**FIGURE 2:**
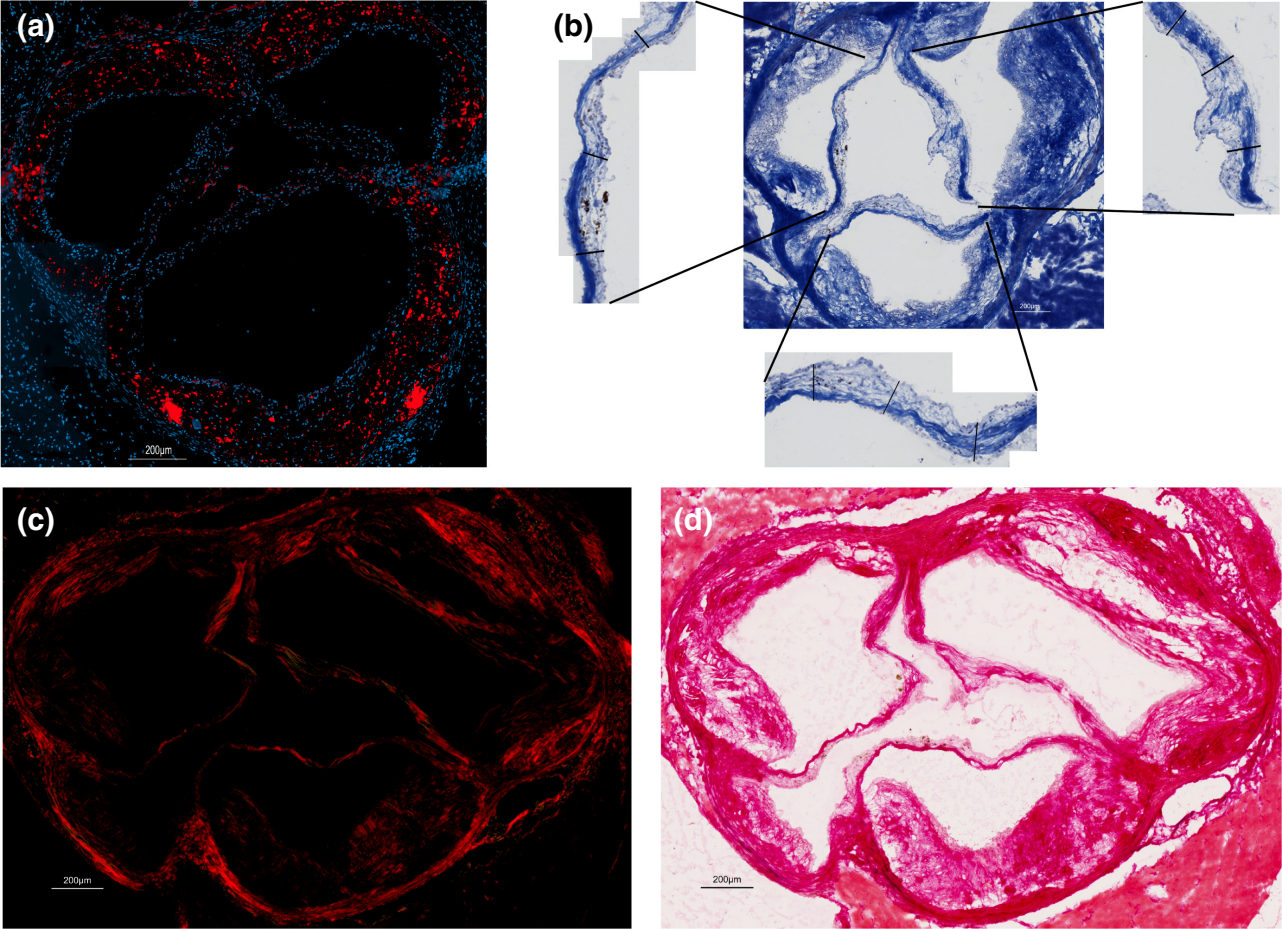
Aortic valve histology methods. (a) Shows the DAPI (4',6‐diamidino‐2‐phenylindole ‐ bleu) coloration of cell nucleus and the OsteoSens* fluorescence (red) of the calcium deposits. (b) Shows the Trichrome Masson staining and the measurement of each leaflet. (c and d) Show the Picrosirius red staining under polarized (c) and white (d) light.

A portion of the mice were sacrificed at 12 weeks (n = 72) and the remaining portion at 36 weeks (*n* = 110). The heart of every mice was harvested. Half of the harvested hearts were embedded in OCT® and 6 μm sections of the aortic valves were obtained by a skilled operator using a cryotome. Histological sections were analyzed with OsteoSense 680EX, Masson's Trichrome, and Picrosirius Red stainings (Appendix S1). All sections were fixed in acetone‐methanol (60:40) at −20°C for 10 min and washed with running tap water for 5 min. All staining kits were obtained from Sigma‐Aldrich Corp (ON, Canada). OsteoSense 680EX staining allowed to assess the presence/absence of valve calcification and quantify the calcification by fluorescence. OsteoSense 680EX staining was quantified with Image J® and presented as the ratio of calcified (red)/total leaflet surface obtained with Dapi (blue) as show in Figure 2(a). Maximal thickness of each cups was measured in sections stained with the Masson's trichrome coloration. Three measurements per cusp were realized and averaged (Figure 2b). Picrosirius red staining was used to study, under polarized light (Figure 2c), collagen fibers (red/orange/green). The amount of collagen fibers (i.e., fibrosis) was expressed as the ratio of polarized pixels over global brightfield tissue pixels (under white light ‐Figure 2d). Picrosirius red staining was analyzed with a custom algorithm developed with MathWorks's MATLAB software detecting pixel RGB values for color differentiation.

### 2.5 Digital droplet PCR (ddPCR)

From the other half of the harvested hearts, aortic roots (from annulus to sino‐tubular junction) containing the aortic valve, were isolated. RNA from the valve was extracted using RNeasy® Mini Kit (Qiagen, ON, Canada). Then PCR were performed with the use of the QX200 Droplet Digital PCR® system. The primers used for the PCR reaction were all from Qiagen (listed below). The Quantasoft software was used to analyze the data. The results of gene expression were normalized with the use of the average of expression of the three following housekeeping genes: hypoxanthine‐guanine phosphoribosyltransferase (HPRT1), Beta‐Actin, and Glyceraldehyde 3‐phosphate dehydrogenase (GAPDH).

Primers used:

- BMP2: QT00012544 (Qiagen, Hilden, Germany)
- BCl2: QT00156282 (Qiagen, Hilden, Germany)
- Casp3: QT00260169(Qiagen, Hilden, Germany)
- BGLAP: QT00259406 (Qiagen, Hilden, Germany)
- RUNX2: QT00020517 (Qiagen, Hilden, Germany)
- ALPL: QT00157717 (Qiagen, Hilden, Germany)
- Wnt5a: QT00160958 (Qiagen, Hilden, Germany)
- MMP9: QT00108815 (Qiagen, Hilden, Germany)
- TIMP: forward 5‐tagtgatggttcccctcctc‐3, reverse 5‐tacttgtttgccatttccca‐3
- AGRT1: QT00233548(Qiagen, Hilden, Germany)
- TGFβ2: QT00058233 (Qiagen, Hilden, Germany)
- TNFα: QT00115332(Qiagen, Hilden, Germany)

### 2.6 Statistical analyses

Continuous variables were tested for normality by the Shapiro–Wilk test. Results were expressed as mean ± SD, median [percentile 25–75] as appropriate.

Echocardiographic longitudinal data were evaluated by a two‐way analysis of variance for repeated measurements followed by Tukey post‐hoc tests. For histologic and ddPCR data, differences between groups were evaluated by a two‐way analysis of variance followed by Tukey post‐hoc tests for continuous normally distributed variables; Wilcoxon rank sum test followed by Steel‐Dwass post‐hoc tests for continuous non‐normally distributed. A value of *p* ˂ 0.05 was considered statistically significant. All statistical analyses were performed with SigmaPlot 11.0 (Systat Software) and Prism 8.0 (GraphPad) software.

## 3 RESULTS

Among the 210 mice used in this study, 110 were followed 36 weeks, 72 were sacrificed at 12 weeks and 28 died unexpectedly (operative mortality: *n* = 9, dental malocclusion: *n* = 6, severe eye ulcer, cancer…: *n* = 13).

### 3.1 Echocardiographic progression of AS according to sex and hormones groups measured by Vpeak and AVAi

One hundred and ten (110) mice underwent echocardiographic examinations at both 12 and 36 weeks. As previously reported (Annabi et al., 2020), IF had lower Vpeak than IM at 12 weeks (94.4 ± 11.3 vs. 118.4 ± 21.2 cm/s; *p* < 0.0001). However, Vpeak progression between 12 and 36 weeks was similar between IF and IM (24.2 ± 5.7 vs. 25.8 ± 5.3 cm/s; *p* = 0.68).

**FIGURE 3:**
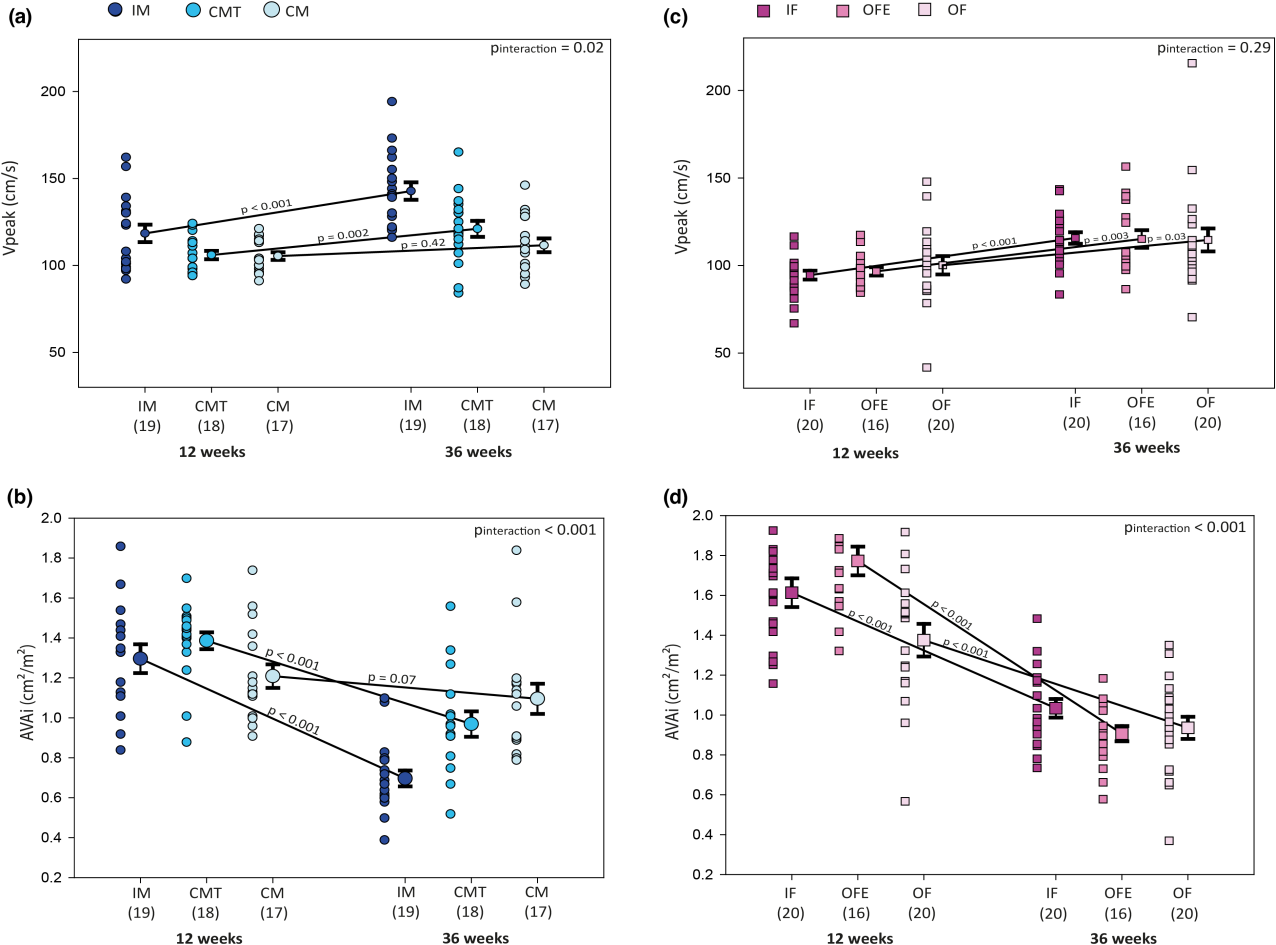
Hemodynamic evaluation of aortic stenosis by echocardiography for each group, at 12 and 36 weeks of age. Changes in peak aortic jet velocity (a and c) and indexed aortic valve area (b and d) between 12 and 36 weeks in males (a and b) and females (c and d) groups. Legend as in Figure 1.

Among male mice (IM *n* = 19; CM *n* = 17 and CMT *n* = 18), there was a significant interaction between hormonal status and time with regards to AS progression (*p* = 0.02). IM had the largest difference in Vpeak between 12 and 36 weeks (24.2 ± 5.7 cm/s; *p* < 0.001) compared to CM (6.2 ± 1.4; *p* = 0.42) who did not progress significantly. Interestingly, CMT had a significant intermediate progression of Vpeak (15.1 ± 3.5; *p* = 0.002, Figure 3a). Accordingly, AVAi decreased significantly in the IM and CMT groups, while the decrease in AVAi was smaller and did not reach statistical significance in the CM group (IM: −0.68 ± 0.21 cm^2^/m^2^; *p* < 0.001 vs. CMT: −0.40 ± 0.20 cm^2^/m^2^; *p* < 0.001 vs. CM: −0.15 ± 0.28 cm^2^/m^2^; *p* = 0.07; *p* for interaction <0.001, Figure 3c).

Among female mice (IF *n* = 20, OF *n* = 20 and OFE *n* = 16), Vpeak progression was significant and similar in all groups (IF: 21.8 ± 5.3; *p* < 0.001; OF: 12.4 ± 2.6; *p* = 0.03; OFE: 18.5 ± 3.4; *p* = 0.003; *p* for interaction = 0.29, Figure 3b). Accordingly, AVAi decreased in the three female groups (IF: −0.60 ± 0.22 cm^2^/m^2^; *p* < 0.001, OFE: −0.85 ± 0.19 cm^2^/m^2^; *p* < 0.001, OF: −0.43 ± 0.22 cm^2^/m^2^; *p* < 0.001; *p* for interaction<0.001, Figure 3d).

Fractional shortening was not different between groups (all *p* > 0.60) and remained stable during the study (*p* = 0.68). Interestingly, cardiac index decreased significantly between 12 and 36 weeks in all groups except CM (CM: *p* = 0.76; all other groups *p* < 0.003, Figure S1).

### 3.2 Evaluation of aortic valve thickness, calcification and fibrosis deposit at 36 weeks

**FIGURE 4:**
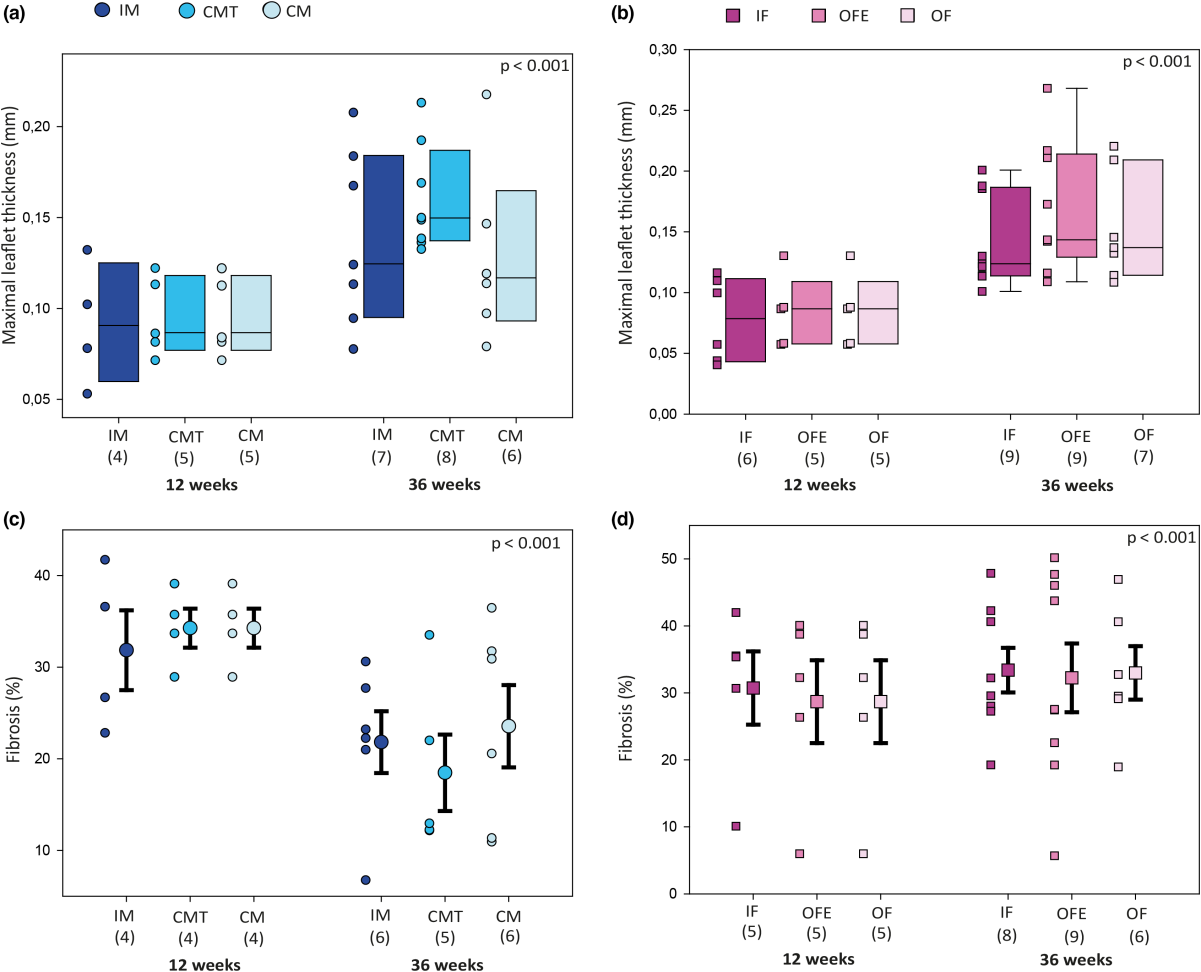
Aortic valve staining presenting valve thickness and fibrosis for each group, at 12 and 36 weeks of age. Average maximal thickness of each aortic valve leaflet was evaluated after Masson Trichrome staining in each group of male (a) and female (b) mice. Percentage of fibrosis was evaluated by red picrosirius staining in each group of male (c) and female (d) mice. Legend as in Figure 1.

Overall, the aortic valve leaflets were thicker at 36 weeks compared to 12 weeks (median [IQR]: 0.141[0.114–0.186] vs. 0.087[0.058–0.113] mm; *p* < 0.001). There were no statistical differences in valve thickness at 36 weeks between all groups, though the CM group had the lowest median valve thickness (0.117[0.093–0.165] mm) (Figure 4a,b).

Compared to valves harvested at 12 weeks, the percentage of fibrosis at 36 weeks appeared to be lower in valves harvested from males while higher in valves harvested from females. Among the three different groups of males, the group with the largest decrease of fibrosis percentage was CMT (from 34.26 ± 4.26 to 18.47 ± 9.32%), compared to IM (from 31.86 ± 8.72 to 21.82 ± 8.27%) and CM (from 34.26 ± 4.26 to 23.56 ± 11.00%, Figure 4c). Among the three groups of females, the increase in fibrosis percentage was similar between all groups (OF: from 28.69 ± 13.82 to 32.98 ± 9.77%, IF: from 30.73 ± 12.22 to 33.39 ± 9.43%, and OFE: from 28.69 ± 13.82 to 32.24 ± 15.40, Figure 4d).

At 12 weeks, we found no evidence of leaflet calcification. At 36 weeks, female mice had lower amounts of calcification than male mice (IF: 0.00[0.00–0.43]%, OF: 0.40[0.17–1.38]%, OFE: 0.45[0.07–1.85]%, IM: 6.01[1.87–40.7]%, CMT: 4.00[0.00–7.05]%, CM: 1.66[0.06–21.80]%; *p* = 0.02). In subgroup analysis, only IF were statistically different from IM (*p* = 0.01).

### 3.3 Up/downregulated pathways in aortic root according to sex and sex hormones

**FIGURE 5:**
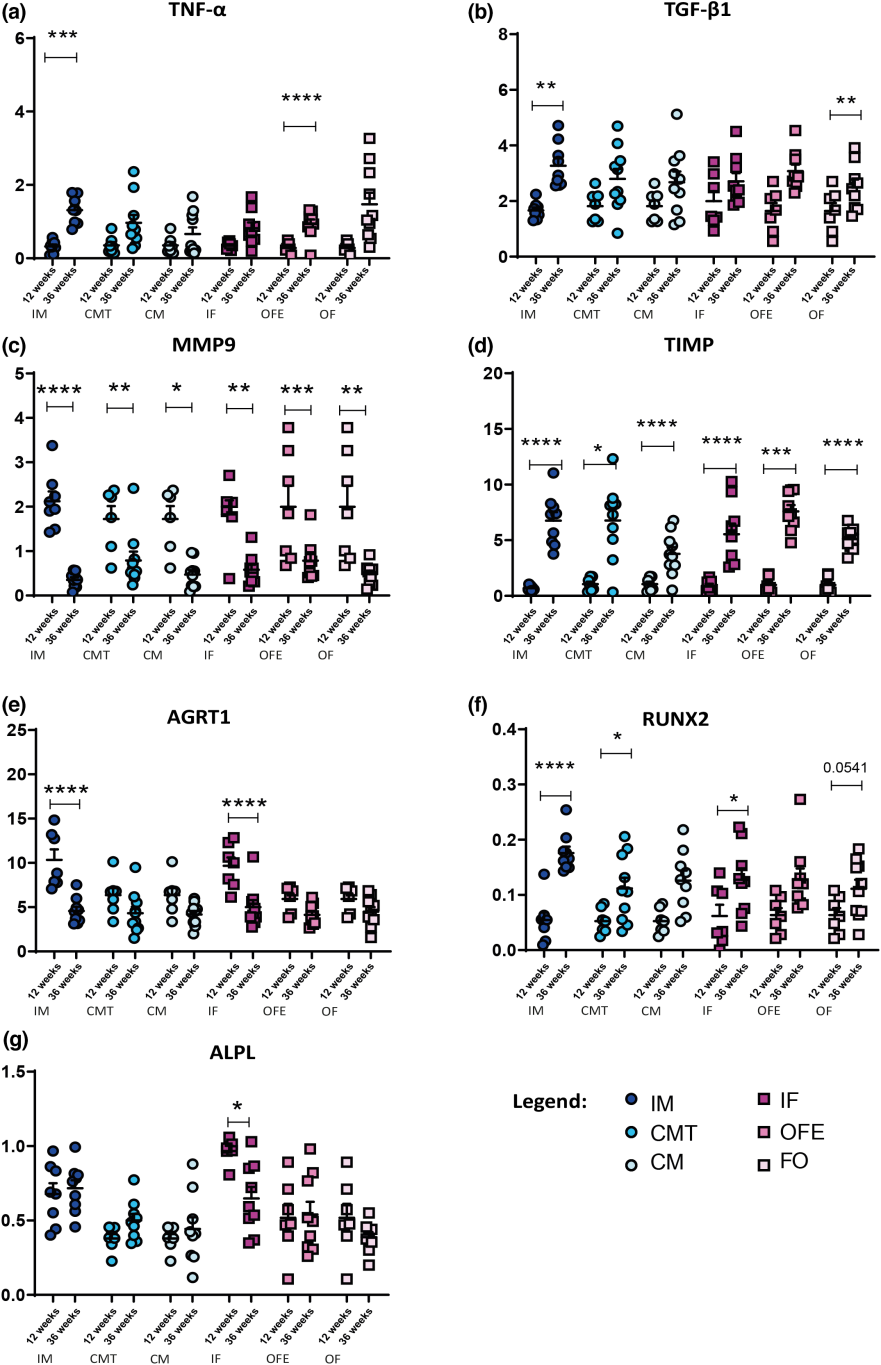
Digital droplet PCR results for gene expression assays of aortic valves RNA samples from each group, at 12 and 36 weeks. (a) shows Tumor Necrosis Factor alpha (TNF‐α), (b) Transforming Growth Factor beta 1 (TGF‐β1), (c) Matrix Metallopeptidase 9 (MMP9), (d) Tissue Inhibitors of Metalloproteinases (TIMP), (e) Angiotensin II receptor type 1 (AGRT1), (f) Runt‐related Transcription factor 2 (RUNX2) and (g) Alkaline Phosphatase (ALPL). Legend as in Figure 1.

Overall, tumor necrosis factor alpha (TNF‐α) transcription was increased at 36 weeks (*p* < 0.0001). However, the increase was important and statistically significant only in IM and OFE (both *p* < 0.001, Figure 5a). The transcription of transforming growth factor beta 2 (TGF‐β2) was upregulated in all groups at 36 weeks (*p* < 0.0001). Despite reaching statistical significance for IM and OF, no interaction was noted between sex/hormone groups and follow‐up duration (*p* = 0.60, Figure 5b). Expressions of matrix metallopeptidase 9 (MMP9) and β‐catenin decreased between 12 and 36 weeks in all groups (both *p* < 0.0001). Downregulation was statistically significant in all groups for MMP9 (Figure 5c) and only in the IF group for β catenin. Accordingly, the expression of metallopeptidase inhibitor (TIMP) increased in all groups during follow‐up (*p* < 0.0001, Figure 5d). Angiotensin II receptor type 1 (AGRT1) expression was decreased at 36 compared in both intact groups (*p* < 0.0001, Figure 5e). Its expression was upregulated in intact groups at 12 weeks compared to gonadectomized ones (*p* = 0.003). There was a statistically significant interaction between sex/hormone groups and follow‐up duration in regards to AGRT1 expression (*p* = 0.02).

When all animals are considered, Runt‐related transcription factor 2 (RUNX2) was upregulated at 36 weeks (*p* < 0.0001). IM had the highest increase at 36 weeks (*p* < 0.0001) and thus, Runx 2 was more upregulated in IM than in other groups (Figure 5f). Expression of alkaline phosphatase (Alpl) was higher in IM and IF at 12 weeks compared to all other groups. It remained high at 36 weeks only in IM while transcription decreased in IF (Figure 5g). Wnt‐5a appeared to be downregulated at 36 weeks (*p* < 0.0001), reaching statistical significance in IF (*p* < 0.05) and with a borderline interaction between groups and follow‐up (*p* = 0.09). The expressions of bone morphogenetic protein 2 (BMP‐2) and B cell lymphoma 2 genes (Bcl‐2) were 10‐fold higher than housekeeping genes in all groups at both follow‐ups (Figure S2).

The expression of Caspase 3 gene (CASP3) was twofold higher than housekeeping genes in all groups at both follow‐ups. However, there was no difference in transcription levels between groups for this gene (Figure S2). Transcription of osteocalcin (BGLAP) was not increased and did not change between 12 and 36 weeks (Figure S2).

## 4 DISCUSSION

In our LDLr^−/−^/ApoB^100/100^/IGF‐II^+/−^ mice, we demonstrated that (1) the progression and severity of AS were influenced by sex and in male mice by the testosterone status; (2) the nature of the stenosed aortic valve lesions were different between male and female mice with more calcification deposits in males and fibrotic remodeling in females; and (3) the modulation of key AS pathogenic pathways were also sex specific with upregulation of pro‐inflammatory genes in IM compared to IF, increased expression of pro‐fibrotic genes in all groups, but lower activation of pro‐calcific genes in female mice compared to IM (Table 1).

**TABLE 1:** Summary of changes in gene expression between 12 and 36 weeks

Gene	Between 12 and 36 weeks		Action of modulation of the gene
	Expression	Group with changes (* largest change)	
Fibrosis			
MMP9	˅	all	Less degradation of the extracellular matrix
TIMP	˄	all	Inhibition of MMPs, thus less degradation of the extracellular matrix
TNFα	˄	all IM* OFE*	Inflammatory cytokine associated with increased fibrosis
TGFβ1	˄	all	Central mediator of increased fibrosis
AGRT1	˅	all IM* IF*	Decreased angiotensin II receptor 1, thus decreased fibrosis
Calcification			
RUNX2	˄	all IM*	Increased osteoblastic differentiation
Alpl	–	Decreased in IF*	Increased bone mineralization
BMP2	–	–	Bone morphogenic factor 2, associated with bone and cartilage formation
BGLAP	–	–	Gene coding for osteocalcin, bone formation marker
Wnt‐5a	˅	all IF*	Implicated in the non‐canonical Wnt pathway associated with calcification
Apoptosis			
BCl2	–	–	Regulates apoptosis
Casp3	–	–	Part of the caspase family which induces apoptosis

### 4.1 Comparison between male and female LDLr^−/−^/ApoB^100/100^/IGF‐II^+/−^ mice

At both 12 and 36 weeks, female mice presented with a less severe stage of AS compared to male mice, which is concordant with female patients whom appear to develop AS at an advanced age. (Fuchs et al., 2010) However, the hemodynamic progression of AS was similar between male and female mice, as seen in clinical patients with AS. (Cramariuc et al., 2015; Tastet et al., 2017) The progression of calcification of the aortic valve was greater in male mice compared to female mice. The degree of AS at the end of the study could be considered moderate, especially in female mice which may explain the low amount of calcium present in the valves in addition to the sex differences. Thus at 36 weeks, calcification was more prevalent in valves explanted from male mice, while female mice had more fibrosis. This is also found in valves explanted during aortic valve replacement surgery in men and women. (Simard et al., 2017; Voisine et al., 2020) Interestingly, as demonstrated in humans, the level of fibrosis seems to decrease in the aortic valve of male mice, as it was replaced by calcification, given that fibrosis‐related genes (TNF‐α and TGF‐β) are also activated in males and thus fibrosis content should be increased. In females, the osteogenic genes, Wnt‐5a and ALPL, were downregulated during follow‐up, suggesting a protection against calcification especially in IF. This downregulation of ALPL may compensate for the slight upregulation of RUNX2 in IF, explaining the small amount of calcification in the valves. On the opposite, RUNX2 was upregulated in IM and CMT indicating an activation of pro‐calcifying pathways associated with the presence of testosterone. In all groups, the massive downregulation of the MMP9 gene and the upregulation of the TIMP gene probably reflected the activation of a negative retro control mechanism linked to fibrosis deposition and matrix turnover, as activation of MMPs and inhibition of TIMPs have been identified as promoters of fibrosis and mineralization in AS (Shen et al., 2017).

Another component of AS progression is apoptosis leading to the calcification of the leaflets. TNF‐α, a gene implicated in cell apoptosis, was increased with disease progression in all groups. TGF‐β, (Jian et al., 2003) a gene that upregulates TNFα was also increased with time in all groups. Despite previously identified as the sex‐specific pathway in AS (Shah & Rogers, 2018), the impact of sex was not significant in BMP/TGF‐β signaling. Other genes related to apoptosis (BCl2 and Casp3) were not influenced by sex, 10‐fold upregulation compared to housekeeping genes equivalent in both sexes, since the beginning of the stenosis processes.

### 4.2 Impact of testosterone in male mice

A previous study demonstrated that androgens induced calcification in vascular smooth muscle cells, and identified the presence of androgen receptor expression in calcified human aortic valve tissue (Zhu et al., 2016). In the present study, testosterone appears to play a major role in AS progression in male mice. Indeed, IM mice had the highest hemodynamic progression of AS while the hemodynamic evaluation of AS in CM mice did not significantly progress. Interestingly, castrated males supplemented with testosterone had an AS hemodynamic progression comprised between the one of CM and intact mice. This impact on hemodynamic progression of AS seems to be triggered by calcification deposits that were found to be fourfold higher in IM compared to CM, and intermediate in CMT, although without statistical significance. This finding is supported by the upregulation of RUNX2, an important gene leading to the mineralization of the aortic valve, in whom the largest activation was found in IM mice.

### 4.3 Impact of estrogen in female mice

When comparing the three female groups, IF mice appeared to differentiate from OF mice with or without 17‐β estradiol. Indeed, valves of IF mice showed a higher downregulation in ALPL and AGRT1, while OF and OFE groups were comparable. Thus, estrogen may only have a minimal role in the pathophysiology of aortic valve in mice.

### 4.4 Limitations of the study

The use of ovariectomy to suppress estrogen does not recapitulate natural menopause which is associated with adaptive compensatory responses. Nevertheless, ovariectomy is the gold standard to evaluate gonadal hormonal effect in female animals. The intact animals did not undergo sham procedure. Thus, the stress caused by the procedure may have influenced results between intact and operated animals. However, this point does not impact results between intact animals as well as between operated animals with and with hormonal supplementation.

The use of percentage to quantify the histologic finding may be confusing as changes could be perceived as small. However, the thickness of the valve (almost double in all groups except CM) needs to be considered to evaluate the total amount of changes in valve composition.

## 5 CONCLUSION

In this murine model of AS, we show that male sex and testosterone play an important role in the calcification of the aortic valve and the hemodynamic progression toward AS. However, female mice appear to be protected against calcification, characterized by downregulation of pro‐osteogenic genes, but present a similar AS hemodynamic progression. Further studies are required to further define the impact of sex hormones in female and the underlying mechanisms leading to the similar progression of AS but sex‐specific fibro‐calcifying process.

## AUTHOR CONTRIBUTIONS

Marie‐Ange Fleury: Work on animal model, analyze the data, build the table/figures of the manuscript and revise it. Mohamed‐Salah Annabi: Perform and analyze echocardiographic examinations, interpret the data and revise the manuscript. Martine Voisine: Work on animal model and laboratory analyses, analyze the data, revise the manuscript. Maxime Hervault: Work on animal model and laboratory analyses, revise the manuscript. Anne‐Julie Boilard: Work on animal model and laboratory analyses, revise the manuscript. Mylène Shen: Work on animal model, revise the manuscript. André Marette: Interpret the data and revise the manuscript. Nancy Côté: Make substantial contributions to the design of the work, interpret the data and revise the manuscript critically for important intellectual content. Marie‐Annick Clavel: Design the study, secure the funding, analyze and interpret the data, draft the article and revise it.

## FUNDING INFORMATION

The study was funded by a grant from the foundation de l'Institut Universitaire de Cardiologie et de Pneumologie de Québec, a grant from the foundation of Universit Laval, and by a Grant‐in‐aid (G‐18‐0022132) from the Heart and Stroke Foundation of Canada. Dr. Clavel holds a New National Investigator Scholarship from the Heart and Stroke Foundation of Canada. Dr. Marette holds a CIHR/Pfizer research Chair in the pathogenesis of insulin resistance and cardiovascular diseases.

## CONFLICT OF INTEREST

None.

## Supporting information


Appendix S1
