# Supplementary material for: Impact of sex and sex hormones on pathophysiology and progression of aortic stenosis in a murine model
Source: Physiol Rep. 2022 Aug 27;10(16):e15433. doi: 10.14814/phy2.15433 (PMC9419154; doi:10.14814/phy2.15433)

Online supplement for

Impact of sex and sex hormones on pathophysiology and progression of aortic stenosis in a murine model

Hormones measurement:

Before sacrifice, blood samples were obtained from all mice. Testosterone was measured in intact males and in castrated males supplemented by testosterone. 17β-estradiol was measured in intact females and ovariectomized females supplemented by 17β-estradiol.

The level of hormones was comparable between intact animals and supplemented animals:

Table: Blood level of testosterone and 17β-estradiol

	IM	CMT		IF	OFE	p-value
Testosterone (ng/ml)	2.65 ±1.38	2.12 ±0.89		-	-	0.42
17β-estradiol (pg/ml)	-	-		5.34 ±2.50	5.10 ±3.70	0.88

Figure S1: Echocardiographic evaluation of LV function

Panels A and B show the cardiac index and **Panels C and D** the fractional shortening at 12 and 36 weeks in male (**Panel A and C**) and female (**Panel B and D**) mice

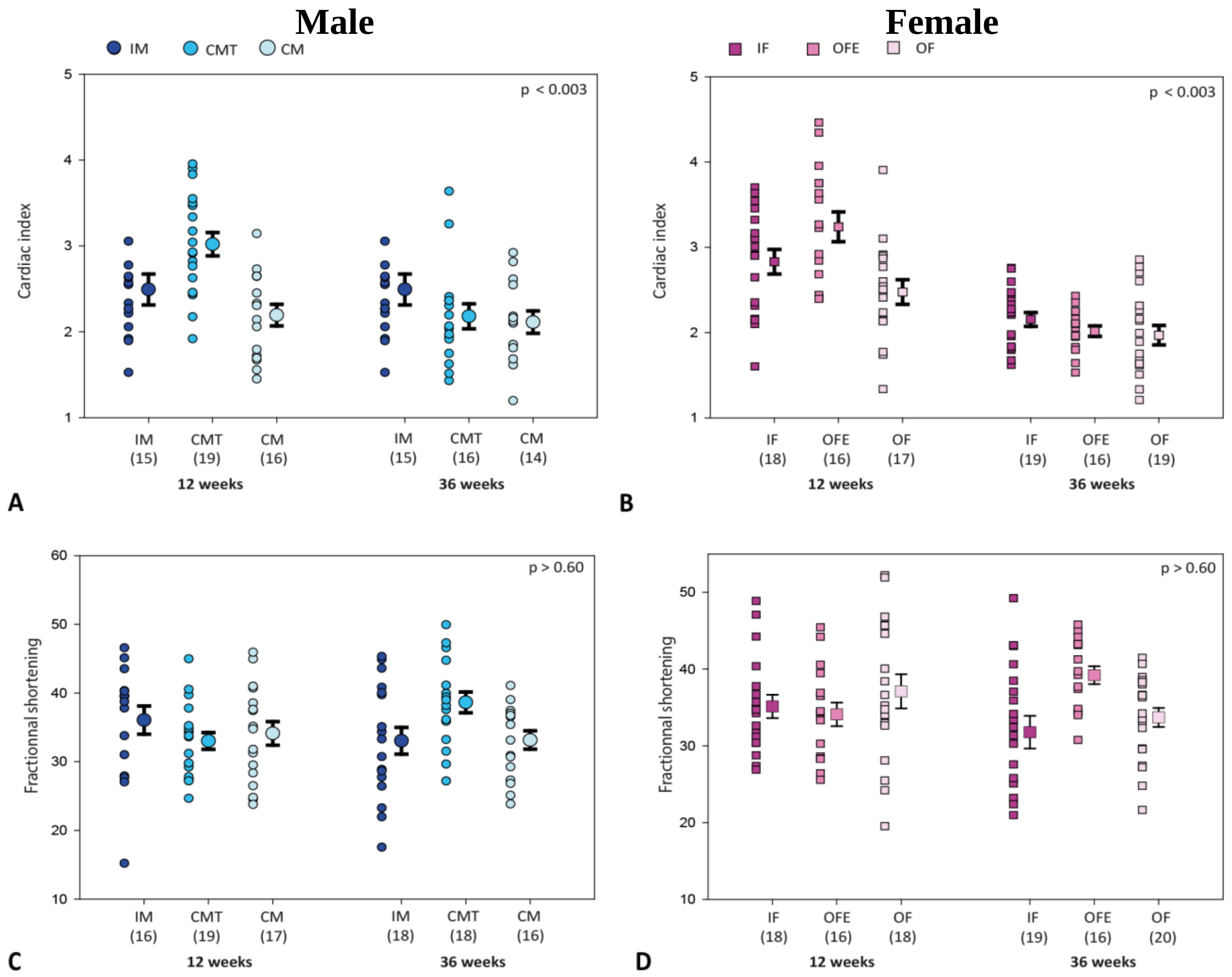

Figure S2: Digital droplet PCR results for gene expression assays of aortic valves RNA samples from each group, at 12 and 36 weeks

Panel A shows Bone morphogenetic protein 2 (BMP2), **Panel B** B-cell lymphoma 2 (BCL2), **Panel C** Caspase 3 and **panel D** bone gamma-carboxyglutamate protein (BGLAP) genes

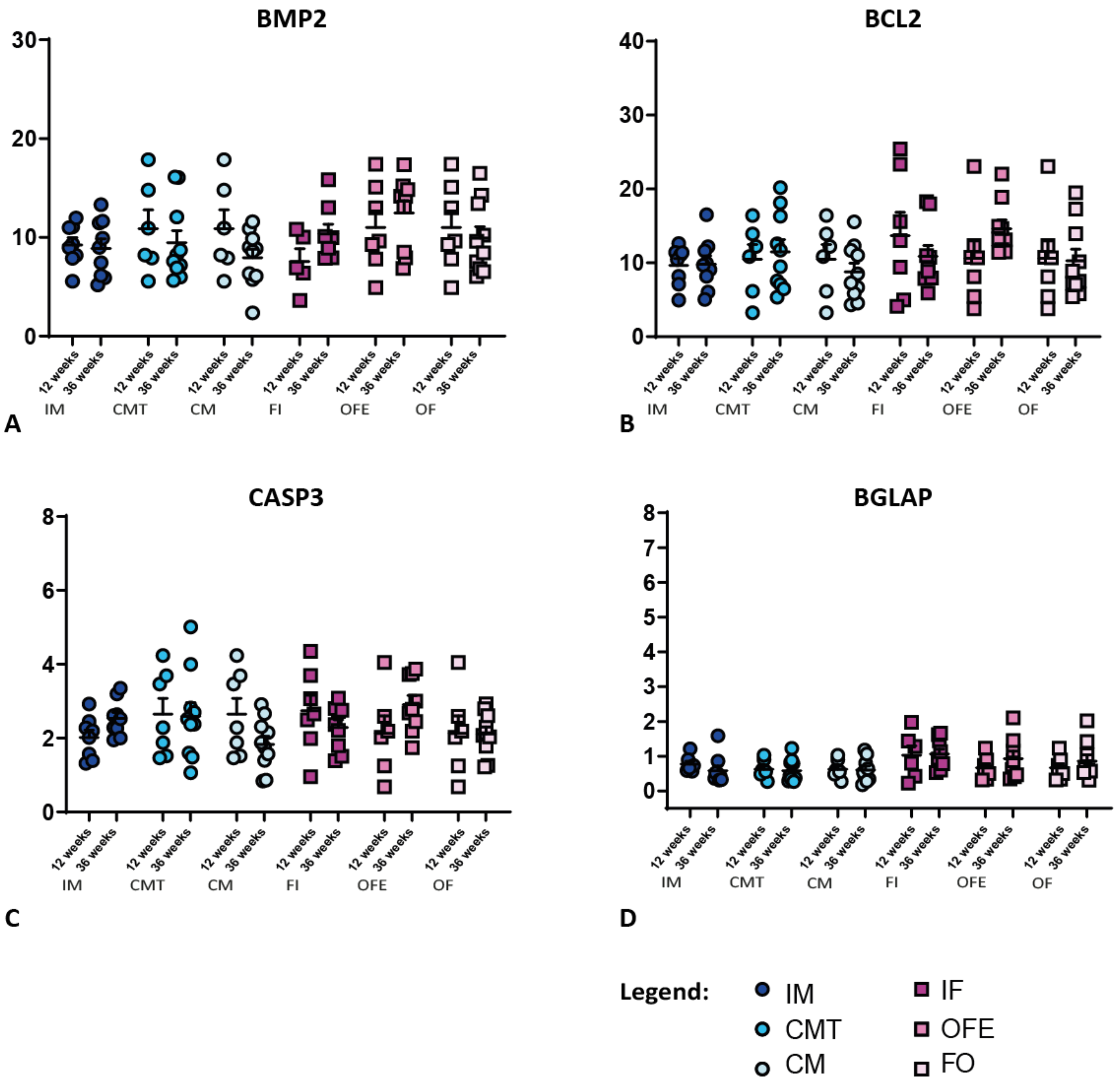

Supplement: Supplementary file 1 — Appendix S1 [file PHY2-10-e15433-s001.pdf]
